# Association of the client-provider ratio with the risk of maternal mortality in referral hospitals: a multi-site study in Nigeria

**DOI:** 10.1186/s12978-018-0464-0

**Published:** 2018-02-22

**Authors:** Friday Okonofua, Lorretta Ntoimo, Rosemary Ogu, Hadiza Galadanci, Rukiyat Abdus-salam, Mohammed Gana, Ola Okike, Kingsley Agholor, Eghe Abe, Adetoye Durodola, Abdullahi Randawa, The WHARC WHO FMOH MNCH Implementation Research StudyTeam

**Affiliations:** 1 University of Medical Sciences, Ondo City, Ondo State Nigeria; 2 the Women’s Health and Action Research Centre, WHO Implementation Research Group, Benin City, Nigeria; 3 Centre of Excellence in Reproductive Health Innovation, University of Benin, Benin City, Nigeria; 4 Department of Demography and Social Statistics, Federal University Oye-Ekiti, Oye, Ekiti State Nigeria; 5 Department of Obstetrics and Gynaecology, University of Port Harcourt, Port Harcourt, Rivers State Nigeria; 6 Aminu Kano Teaching Hospital, Kano, Nigeria; 7 Adeoyo Maternity Hospital, Ibadan, Oyo State Nigeria; 8 General Hospital, Minna, Niger State Nigeria; 9 Karshi General Hospital, Federal Capital Territory, Abuja, Nigeria; 10 Central Hospital, Warri, Delta State Nigeria; 11 Central Hospital, Benin City, Edo State Nigeria; 12 General Hospital, Ijaye Abeokuta, Ogun State Nigeria; 13 Ahmadu Bello University, Zaria, Kaduna State Nigeria

**Keywords:** Client-provider ratio, Maternal mortality, Nigeria, Referral hospitals

## Abstract

**Background:**

The paucity of human resources for health buoyed by excessive workloads has been identified as being responsible for poor quality obstetric care, which leads to high maternal mortality in Nigeria. While there is anecdotal and qualitative research to support this observation, limited quantitative studies have been conducted to test the association between the number and density of human resources and risk of maternal mortality. This study aims to investigate the association between client-provider ratios for antenatal and delivery care and the risk of maternal mortality in 8 referral hospitals in Nigeria.

**Methods:**

Client-provider ratios were calculated for antenatal and delivery care attendees during a 3-year period (2011–2013). The maternal mortality ratio (MMR) was calculated per 100,000 live births for the hospitals, while unadjusted Poisson regression analysis was used to examine the association between the number of maternal deaths and density of healthcare providers.

**Results:**

A total of 334,425 antenatal care attendees and 26,479 births were recorded during this period. The client-provider ratio in the maternity department for antenatal care attendees was 1343:1 for doctors and 222:1 for midwives. The ratio of births to one doctor in the maternity department was 106:1 and 18:1 for midwives. On average, there were 441 births per specialist obstetrician. The results of the regression analysis showed a significant negative association between the number of maternal deaths and client-provider ratios in all categories.

**Conclusion:**

We conclude that the maternal mortality ratios in Nigeria’s referral hospitals are worsened by high client-provider ratios, with few providers attending a large number of pregnant women. Efforts to improve the density and quality of maternal healthcare providers, especially at the first referral level, would be a critical intervention for reducing the currently high rate of maternal mortality in Nigeria.

**Trial registration:**

Trial Registration Number: NCTR91540209. Nigeria Clinical Trials Registry. Registered 14 April 2016.

## Plain English summary

The considerable number of women who die during pregnancy in Nigeria is currently a critical health concern. This paper addresses the question of whether the high number of women dying in pregnancy in Nigeria’s referral hospitals might be associated with the inadequate number of healthcare providers (doctors, midwives and nurses) who attend large numbers of pregnant women.

The total number of healthcare providers (doctors, specialist doctors, nurses and midwives) working in eight referral hospitals during the period was calculated. The number of women who attend antenatal and delivery care in the hospitals was also calculated. We then calculated the ratio of the number of pregnant women attending antenatal and delivery care to the number of different categories of healthcare providers (doctors, nurses, midwives and specialist doctors). Finally, we performed additional statistical analysis and examined the relationship between the number of women who died during childbirth in hospitals and the ratio of clients per available healthcare provider. The results showed that the higher the ratio of clients/patients who were managed by doctors, nurses, midwives and specialist obstetricians and gynaecologists during antenatal and delivery care, the higher the maternal mortality ratio in the hospitals.

We conclude that the number of women who die during childbirth in Nigeria’s referral hospitals is associated with the low number of healthcare providers. Efforts to increase the number of trained maternal healthcare providers, especially at the first referral level, is essential for reducing the currently high rate of maternal deaths in Nigeria.

## Background

The high rate of maternal mortality in Nigeria is currently one of the most critical public health concerns in the country. The available data indicate that approximately 40,000 women die each year from preventable causes, with a maternal mortality rate of 814/100,000 births [1], accounting for 15% of the annual global estimates of maternal deaths [2]. Several factors have been offered to explain the high rate of maternal mortality in Nigeria. These include high rates of unwanted and mistimed pregnancies [3, 4], delays in treatment seeking pregnancy care [5, 6], low use of skilled birth attendants [7–9], and inadequate preparedness of the healthcare system to handle emergency obstetric complications [10–13].

However, to date, the readiness and adequacy of human resources in the country to address obstetric complications that lead to maternal mortality have not been systematically investigated. It is generally believed that the paucity of human resources and lack of training and motivation buoyed by excessive workloads are responsible for poor quality obstetric care that leads to maternal deaths in women with pregnancy complications in hospitals. While there is anecdotal and qualitative research to confirm these assertions in many parts of Africa [14–18], limited quantitative studies have been conducted to test the association between the quantum of human resources and risk of maternal mortality.

The Nigerian healthcare system established primary healthcare centres to offer Basic Emergency Obstetric Care (BEmOC) according to the recommendations of the World Health Organization and to treat simple obstetric complications that do not warrant surgical operations. By contrast, secondary and tertiary care centres were established to offer Comprehensive Emergency Obstetric Care (CEmOC) to provide additional treatments, including blood transfusions and surgical operations. If the system works well, approximately 70% of emergency complications would be handled at the level of BEmoC, while only a limited fraction would seek CEmOC. However, due to the inadequate functioning of the country’s primary healthcare system, most emergencies are often referred for CEmOC in referral hospitals, thereby increasing the workloads and limiting the quality of care provided in secondary and tertiary hospitals. With this limitation in mind, the relevant question is whether increased human resource provision at referral hospitals would have a restraining effect on the rates of maternal mortality in the country.

This study was designed to investigate the association between the density of healthcare providers (doctors, nurses and midwives) and risk of maternal deaths in Nigeria’s referral hospitals. We tested the null hypothesis that an increased number of healthcare providers would have no effect on the maternal mortality ratios by investigating the density of healthcare personnel in eight referral hospitals and comparing it with the maternal mortality ratios in the hospitals. We believe that the results of this study will be useful for planning the allocation of the scarce human resources available for healthcare to address the high rate of maternal mortality in Nigeria.

## Methods

The data for this study were obtained as part of the formative research for a study on assessing the nature and quality of emergency obstetric care for preventing maternal and perinatal mortality in eight secondary and tertiary hospitals in Nigeria. Nigeria’s healthcare system is based on a 3-tier system: Primary Health Care (PHC) as the entry point; Secondary Care (regional/general hospitals) as the first referral level; and Tertiary Care (Teaching Hospitals) as the second referral level. In this study, eight referral hospitals (two tertiary and six secondary facilities) were selected from four of six geo-political zones of Nigeria. Administratively, Nigeria has 36 states and a Federal Capital Territory (Abuja). The states are further categorized into six zones: North-central, Northeast, Northwest, Southeast, South-south, and Southwest, with each zone predominantly consisting of people of a similar culture. Two tertiary hospitals were selected from the Northwest: Ahmadu Bello University Teaching Hospital, Kaduna and Aminu Kano Teaching Hospital, Kano. Secondary care facilities were selected from three other zones: General Hospital, Ijaye, Abeokuta and Adeoyo Maternity Hospital in the Southwest; General Hospital, Minna, Niger State and Karshi Hospital, Abuja in the North-central; and Central Hospital, Benin City and Central Hospital, Warri from the South-south. We did not select any referral hospitals from the Northeast zone because it is difficult to reach these hospitals because of the ongoing insurgency in the zone. To counterbalance the selection, we also failed to sample hospitals in the contiguous southeast zone. The hospitals that we selected were selected because they are the main referral hospitals in the respective states; they attend a large population of pregnant women in the main cities of the states, with none having fewer than 4500 total deliveries per year. A large proportion of women presented in labour at the hospitals as obstetric emergencies who had intended to deliver at home, in private clinics or in the homes of traditional birth/faith-based birth attendants, but then came late to the hospitals when they experienced complications. These types of deliveries constitute approximately 50% of cases in the hospitals, accounting for an average of 150 obstetric emergencies per month per hospital. All of the hospitals are funded either by the respective states or the federal government, which means that the hospital staffing policies are also overseen by governmental departments.

Because this was a formative study leading to the design of a larger intervention, we chose pairs of states with catchment populations with similar socio-demographic characteristics to adequately represent a quasi-experimental research design. Thus, we first chose the largest referral hospitals in four states. Based on our knowledge of the ecology of the use of maternal health services, we hypothesized that secondary referral hospitals (General hospitals) have a larger uptake of obstetric emergencies, have fewer healthcare providers, and therefore have increased numbers of maternal deaths. For this reason, we sampled three secondary referral hospitals compared to only one tertiary hospital (teaching hospital). In the second stage of sampling, we chose comparative main referral hospitals in adjoining states different from the states in which the initial selections were made. The purpose was to eliminate the effect of intervention contamination if we implement the intervention at a later stage. We decided to choose teaching hospitals from the northern part of the country because of the reported higher numbers of deliveries and maternal deaths in northern Nigeria hospitals compared to the southern part of the country. The Aminu Kano Teaching Hospital is one of the busiest teaching hospitals in Nigeria, located in Kano State, northern Nigeria, and was identified in the first stage of sampling. For comparison, we then identified the Ahmadu Bello University, Zaria, which is also a busy teaching hospital located in the contiguous state of Kaduna.

Information on the number of doctors, nurses, midwives, clients reporting for antenatal care, number of births and number of maternal deaths were obtained from each hospital for the three-year period (1 January 2011 to 31 December 2013) preceding the formative research, which was conducted between 1 January 2014 to 30 June 2014. Each participating hospital was visited, and a pre-tested study protocol was used to elicit the relevant information regarding the number of healthcare workers, deliveries, and maternal deaths during the period. The numbers of antenatal care attendees, births and maternal deaths were aggregated for the 3 years to derive the average attendees, births and maternal deaths for the period.

### Ethical approval

Ethical approval for the study was obtained from the World Health Organization and the National Health Research Ethics Committee (NHREC) of Nigeria – number NHREC/01/01/2007–16/07/2014, renewed in 2015 with NHREC 01/01/20047–12/12/2015b. The Chief Medical Directors and Heads of Departments of the hospitals women were informed of the purpose of the study, and consent was obtained from the patients to conduct the study. Patients were assured that their information would be confidential. Only the hospitals that agreed to participate in the fully explained study were enlisted in the study. No names or specific contact information were obtained from the study participants.

### Analytical strategy

Ratios was used to express the density of antenatal care (ANC) attendees to a provider and the number of births to a provider. Given that the quality of maternal health care provided in a hospital is a function of contributions from various departments, ratios were calculated for the following seven categories of providers: 1) all providers in the hospital consisting of all doctors in the Department of Obstetrics and Gynaecology, including specialist obstetricians, nurses/midwives, doctors and nurses in other departments in the hospital; 2) all doctors, consisting of doctors in the Department of Obstetrics and Gynaecology, specialist obstetricians, and doctors in other departments; 3) all nurses and midwives, including nurses only, nurses/midwives and midwives in the entire hospital; 4) all providers in the maternity department (mproviders), including doctors in the Department of Obstetrics and Gynaecology, specialist obstetricians, nurses/midwives and midwives; 5) all doctors in the maternity department (mdoctors), including doctors in the Obstetrics and Gynaecology Department and specialist obstetricians; 6) specialist obstetricians only; and 7) trained midwives, including nurses/midwifes and midwives. The maternal mortality ratio (MMR) was calculated per 100,000 live births. Unadjusted Poisson regression analysis was used to examine the association between the number of maternal deaths and number of providers. The Poisson regression models are best suited for count variables, such as the number of deaths. The results are presented as the incidence rate ratio (IRR), with the level of significance set at *p* < 0.05 and the marginal significance set at *p* < 0.10 because of the small sample size. A two-tailed Pearson product moment correlation was used to examine the statistical association between the births-provider ratio, ANC attendee-provider ratio and maternal mortality ratio, and the results are presented as correlation coefficients (r) with the level of significance set at *p* < 0.05. All analyses relating to maternal deaths were conducted using data from seven of eight hospitals because data on maternal deaths were not available in Karshi General Hospital, Abuja.

## Results

**Fig. 1:**
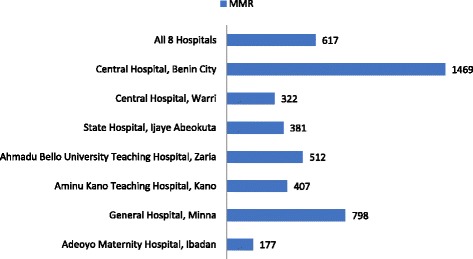
Maternal Mortality Ratios in the Hospitals

The distribution of all providers as percentages, numbers and the average number of maternal deaths is shown in Table 1. The results of the unadjusted Poisson regression of the number of maternal deaths and number of providers are presented in Table 2. The MMR is shown in Fig. 1. Table 3 presents the ratio of births to a provider (births-provider ratio) and average number of births, while the ANC attendees-provider ratio and average number of ANC attendees are shown in Table 4 with the associated Pearson correlation coefficients.

**Table 1:** Percentage Distribution of Health Care Providers and average maternal deaths by Facility

Facility	All providers	All doctors	All nurses & midwives	Doctors in Obs/Gyn	Specialist Obstetricians	mdoctors	Midwives	mproviders	Average mdeath
Adeoyo Maternity Hospital, Ibadan	6.5 (245)	2.9 (40)	8.7 (205)	9.0 (17)	6.7 (4)	8.4 (21)	12.9 (195)	12.3 (216)	3.8 (6)
General Hospital, Minna	9.4 (351)	3.7 (52)	12.6 (299)	4.2 (8)	3.3 (2)	4.0 (10)	11.6 (175)	10.5 (185)	27.2 (43)
Aminu Kano Teaching Hospital, Kano	26.0 (977)	28.8 (400)	24.4 (577)	26.5 (50)	28.3 (17)	26.9 (67)	6.5 (98)	9.4 (165)	13.3 (21)
Ahmadu Bello University Teaching Hospital, Zaria	37.4 (1405)	49.4 (685)	30.4 (720)	28.6 (54)	36.7 (22)	30.5 (76)	42.9 (648)	41.2 (724)	6.3 (10)
State Hospital, Ijaye Abeokuta	5.2 (195)	3.2 (45)	6.3 (150)	5.8 (11)	3.3 (2)	5.2 (13)	5.6 (85)	5.6 (98)	3.2 (5)
Karshi General Hospital, Abuja	1,9 (73)	1.2 (17)	2.4 (56)	7.4 (14)	1.7 (1)	6.0 (15)	2.7 (41)	3.2 (56)	N/A
Central Hospital, Warri	5.3 (198)	4.9 (68)	5.5 (130)	9.0 (17)	6.7 (4)	8.4 (21)	7.7 (117)	7.8 (138)	8.9 (14)
Central Hospital, Benin City	8.3 (310)	5.8 (80)	9.7 (230)	9.5 (18)	13.3 (8)	10.4 (26)	9.9 (150)	10.0 (176)	37.3 (59)
Total	100 (3754)	1387	2367	189	60	249	1509	1758	158

IRR – Incidence Rate Ratio

All providers refer to all doctors, nurses and midwives in the entire hospital; All doctors refer to all doctors in all the departments in the entire hospital; All nurses and midwives refer to all nurses and midwives in the entire hospital; mdoctors refers to doctors in Obstetrics and Gynaecology department and specialists obstetricians; mproviders refers to doctors in Obstetrics and Gynecology Departments, specialist Obstetricians and midwives; mdeath refers to maternal deaths

**Table 2:** Unadjusted Incidence Rate Ratios (and 95% confidence intervals) from Poisson regression analyses examining association between number of providers and number of maternal deaths

Type of provider	IRR	CI
All Providers	0.999**	0.999–1.000
All Doctors	0.998***	0.998–0.999
All Nurses & Midwives	0.999	0.999–1.000
Doctors in Obstetrics & Gynaecology Department	0.986***	0.980–0.992
Specialist Obstetricians	0.985*	0.973–0.998
Trained Midwives	0.998***	0.998–0.999
Doctors in Obstetrics & Gynaecology Department & Specialists Obstetricians	0.992***	0.988–0.996
All maternal providers	0.998***	0.998–0.999

Note: Significance-level (two-tailed): * *p* < .05, **p < .01, ***p < .001. IRR – Incidence Rate Ratio; CI – 95% Confidence Interval

**Table 3:** Births: Providers ratio and average number of births

Hospital	All providers	All doctors	All nurses & midwives	Specialist Obstetricians	mDoctors	Midwives	mProviders	Average births %(n)
Adeoyo Maternity Hospital, Ibadan	14	85	17	850	162	17	16	12.8 (3399)
General Hospital, Minna	15	104	18	2695	539	31	29	20.4 (5389)
Aminu Kano Teaching Hospital	5	13	9	304	77	53	31	19.5 (5164)
Ahmadu Bello University Teaching Hospital, Zaria	1	3	3	89	26	3	3	7.4 (1955)
State Hospital, Ijaye Abeokuta	7	29	9	657	101	15	13	4.9 (1314)
Karshi General Hospital, Abuja	12	52	16	892	59	22	16	3.4 (892)
Central Hospital, Warri	22	64	33	1087	207	37	32	16.4 (4349)
Central Hospital, Benin City	13	50	17	502	155	27	23	15.2 (4017)
All 8 Hospitals	7	19	11	441	106	18	15	26,479
Pearson Correlation Coefficient (r)	0.060	0.080	0.001	0.095	0.228	0.043	0.134	

All providers refer to all doctors, nurses and midwives in the entire hospital; All doctors refer to all doctors in all the departments in the entire hospital; All nurses and midwives refer to all nurses and midwives in the entire hospital; mdoctors refers to doctors in Obstetrics and Gynaecology department and specialists Obstetricians; mproviders refers to doctors in Obstetrics and Gynaecology Departments, specialist Obstetricians and midwives

**Table 4:** ANC Attendees: Providers ratio and average number of ANC Attendees

Hospital	All providers	All doctors	All nurses & midwives	Specialist Obstetricians	mDoctors	Midwives	mProviders	Average ANC attendees %(n)
Adeoyo Maternity Hospital, Ibadan	23	141	28	1413	269	29	26	1.7 (5651)
General Hospital, Minna	28	190	33	4939	988	56	53	2.9 (9877)
Aminu Kano Teaching Hospital	32	77	54	1821	462	316	188	9.3 (30963)
Ahmadu Bello University Teaching Hospital, Zaria	7	15	14	460	133	16	14	3.0 (10118)
State Hospital, Ijaye Abeokuta	91	396	119	8905	1370	210	182	5.3 (17810)
Karshi General Hospital, Abuja	34	145	44	2472	165	60	44	0.7 (2472)
Central Hospital, Warri	162	472	247	8017	1527	274	232	9.6 (32066)
Central Hospital, Benin City	727	2818	980	28,183	8672	1503	1281	67.4 (225467)
All 8 Hospitals	89	241	141	5574	1343	222	190	334,425
Pearson Correlation Coefficient (r)	0.851*	0.870*	0.841*	0.847*	0.890*	0.848*	0.858*	

Note: Significance-level (two-tailed): *p < .05

All providers refer to all doctors, nurses and midwives in the entire hospital; All doctors refer to all doctors in all the departments in the entire hospital; All nurses and midwives refer to all nurses and midwives in the entire hospital; mdoctors refers to doctors in Obstetrics and Gynaecology department and specialists Obstetricians; mproviders refers to doctors in Obstetrics and Gynaecology Departments, specialist Obstetricians and midwives

### Distribution of healthcare providers and the average number of maternal deaths by facility

There was a total of 3754 providers in the 8 hospitals, of which 1387 (36.9%) were doctors and 2367 (63.1%) were nurses and midwives. The maternal healthcare providers (mdoctors and midwives) constituted 46.8% (1758) of all of the providers. The distribution varied widely across facilities. More than half (63.4%) of all providers were in the two teaching hospitals (Aminu Kano Teaching hospital, Kano and Ahmadu Bello University Teaching hospital, Kaduna), while Karshi General Hospital had the least number of providers. The largest number of mproviders (41.2%) was in Ahmadu Bello Teaching hospital, while Karshi General Hospital, Abuja had the smallest number of providers. There was a total of 60 specialist obstetricians in all of the facilities, with only one in Karshi General Hospital, Abuja, 2 in General Hospital Minna and 2 in State Hospital Ijaye, Abeokuta.

On average, 334,425 women received antenatal care in the 8 facilities during the three-year period (Table 4), while an average of 26,479 (Table 3) births occurred over the same period. Expressing the number of births as a percentage of the number of ANC attendees indicates that fewer than 10% of women who received ANC in the 8 facilities delivered in the facilities. However, there were variations across the facilities. For instance, births in Adeoyo Maternity Hospital and General Hospital, Minna occurred for over 50% of the ANC attendees, whereas births in Central Hospital, Benin City occurred for 1.8% of the ANC attendees. Of the 25,587 live births in 7 facilities with a record of maternal deaths, an average of 158 maternal deaths was recorded, giving a maternal mortality ratio of 617 per 100,000 live births. The distribution across the hospitals (Fig. 1) shows that Central Hospital, Benin City recorded the highest MMR of 1469 per 100,000 live births, whereas the smallest MMR was found for Adeoyo Maternity Hospital, Ibadan (177/100,000 live births).

Unadjusted Poisson regression analysis (Table 2) showed a significant negative association between the number of maternal deaths and the number of healthcare providers. The incidence of maternal death would be expected to decrease significantly by a factor of 0.999 (*p* < 0.01) for a unit increase in the number of all providers. An increase in the number of all doctors will decrease the incidence of maternal death by a factor of 0.998 (*p* < 0.001), and an increase in the number of nurses and midwives is expected to decrease maternal death, although with marginal statistical significance (0.999 *p* < 0.10). Increasing the number of doctors in the Department of Obstetrics and Gynaecology who are not specialist obstetricians would significantly decrease the incidence of maternal death (IRR, 0.986; p < 0.001). With a unit increase in the number of specialist obstetricians, the IRR of maternal death would decrease (IRR, 0.985; *p* < 0.05). Increasing the number of all doctors in the maternity department, including specialist obstetricians is expected to decrease maternal death by a factor of 0.992 (*p* < 0.001), and a one-unit increase in the number of midwives will reduce maternal death (IRR, 0.998; *p* < 0.001). Increasing the number of all providers in the maternity department (mproviders) by one unit would significantly decrease maternal death (IRR, 0.998; *p* < 0.001).

#### Births-provider ratio

The ratio of births to all providers was 7:1, with variation across the facilities. The smallest births-all provider ratio was at the Ahmadu Bello Teaching Hospital, Zaria, where the ratio was 1:1, whereas the highest ratio was found in Central hospital, Warri, with 22 births to a provider. The ratio of births to all doctors was 19:1 and 11:1 for all nurses and midwives, respectively. Again, the smallest ratio was in Ahmadu Bello University Teaching Hospital, whereas the highest was in Central hospital, Warri. There were 441 births per specialist obstetrician, for a ratio of 2695:1 in General Hospital, Minna and 1087:1 in Central Hospital, Warri. The ratio of births to one doctor was 106 compared to 18:1 for midwives. Correlating the births to provider ratios to MMR showed a positive statistical association, indicating that as the birth to provider ratio increased, the number of maternal deaths increased; however, the results were not statistically significant.

#### ANC attendee-provider ratio

The ANC attendee to all provider ratio was 89:1, with Central Hospital, Benin City recording the highest ratio of 727 attendees to one provider. The ratio of ANC attendees to a specialist obstetrician was 5574:1; only Ahmadu Bello Teaching Hospital had a ratio below 1000:1. The ratio was 190 attendees to a provider. Disaggregating providers into doctors and midwives showed a ratio of 1343 ANC attendees to a doctor and 222 attendees to one midwife. The smallest ANC attendee-maternal healthcare provider ratio was in Ahmadu Bello Hospital, Zaria, with 14 ANC attendees to an provider, and the largest ratio was in Benin, with 1281 attendees to a provider. Although the births-provider ratio is more critical for maternal mortality, the ANC attendee-provider ratio speaks to the quality of ANC care, which is a risk factor for MMR. If the ratio is high, providers may not be able to provide optimal care, thus exposing more women to the risk of maternal death. The correlation coefficients were all positive and significant, indicating that the higher ANC attendee to provider ratio increased MMR.

## Discussion

The study was designed to investigate the association between the client-provider ratios in Nigeria’s referral hospitals and maternal deaths in the hospitals. We specifically studied how the number and density of maternal healthcare providers in hospitals predict the likelihood of maternal deaths. To the best of our knowledge, this study is one of the first to quantitatively investigate this relationship, especially in regions with high rates of maternal death. Although there is evidence in the global literature that the increased density of human resources (especially nurses, specialists and non-specialist doctors) reduces maternal, infant and child mortality [19, 20], there has been limited specific investigation in many African countries aimed at testing this relationship.

Contrary to our null hypothesis, the results of this study showed that there was a significant negative association between the number of maternal deaths and total number of providers in the hospital. We found that the incidence of maternal death significantly decreases by a factor of 0.985–0.999 with a unit increase in the number of providers of any category. This is not surprising because prevention of maternal death in a referral hospital is the responsibility of all providers working together in different departments, especially the obstetrics and gynaecology, nursing/midwifery, pathological sciences, paediatrics, medicine and surgical departments. The number of maternal deaths also showed a negative correlation with the total number of maternal healthcare providers (doctors and midwives) working in the maternal sections of the hospitals, further emphasizing the importance of a high density of providers in preventing maternal deaths in the referral hospitals.

Pregnant women receiving antenatal and intrapartum care is also a major determinant of survival. It was noteworthy that only approximately 10% of women who initially received antenatal care returned for delivery at the same hospitals. This relationship was particularly severe in the General Hospitals, especially at the Central Hospital, Benin City, fewer than 2% of women who received antenatal care returned for delivery care at the same hospital. It was not surprising, therefore, that the Central Hospital, Benin City had the highest number and ratio of maternal deaths. Although the data were not disaggregated by the booking status of women, it appears that hospitals (especially the Central Hospital) mainly attended women who had not received antenatal care, but who turned up at the time of delivery or when they experienced complications. Although a tertiary referral hospital, the Ahmadu Bello University Teaching Hospital had only 50% of its antenatal attendees return for delivery care, which probably accounted for its lower maternal mortality ratios compared to the other hospitals. Future research is needed to address this bottleneck, especially to understand why women who received antenatal care in a particular hospital failed to return for delivery at the same hospital. Clearly, focusing on ways to retain women who receive antenatal care to deliver at the same hospital is an important intervention for preventing maternal deaths in hospitals.

The results of this study show a positive and significant correlation between a higher ANC attendee to provider ratio and increases in MMR, suggesting that higher numbers of antenatal care providers would significantly reduce the likelihood of maternal mortality. Similarly, although the results were not statistically significant, there was a positive association between higher birth to provider ratios and increases in the ratios of maternal mortality in the hospitals. Increasing women’s access to skilled birth facilities has been identified as an effective intervention to reduce maternal deaths [21, 22] in developing countries. The results of this study suggest that an adequate number of healthcare providers working in referral hospitals is also important if women who seek emergency obstetric care in referral hospitals are to be offered the best chance of survival. The results of this study reveal that all of the indicators of human resources for maternal health (the number of total providers and providers and client-provider ratios) were better for the Teaching Hospitals (Ahmadu Bello University Teaching Hospital and the Aminu Kano Teaching Hospital) compared to General (Central) Hospitals. In Nigeria’s healthcare system, Teaching Hospitals are the second referral level for maternal health care, while General Hospitals are supposed to function as the first referral level. Unfortunately, the system does not work this way as Teaching and General Hospitals are often used simultaneously for both primary care as well to treat complications. Although both types of hospitals perform similar tasks with respect to maternal health care, General Hospitals have not received a concomitant level of funding and human resource support, which accounts for the poorer human resource outcomes reported for these hospitals. Thus, we believe that if positive results are to be obtained for reducing maternal deaths in women referred for comprehensive emergency obstetric care in Nigerian referral hospitals, efforts must be made to improve the human resource situation in General Hospitals.

The results of this study are consistent with the findings of our previous qualitative studies [23], which reported that women attending these hospitals perceived that maternal mortality and poor obstetric outcomes in these hospitals are due to heavy workloads experienced by healthcare providers. Our recent study also reported long wait and contact times experienced by women receiving antenatal care in these hospitals [24], which is indicative of the diminished numbers of healthcare providers attending women in the hospitals. Thus, we believe that efforts focused on increasing the number of healthcare providers in maternal care centres would improve the quality of care and reduce the maternal mortality ratios.

The major strength of this study is the use of data from hospitals in four of the six geo-political zones of the country. We were unable to obtain data from the Northeast zone of the country because of the on-going insurgency, which limited our access to referral hospitals in that part of the country. We believed that the results from the Northeast zone would be difficult to compare with the other zones in the country because of the likelihood that women would be self-selective in receiving maternal health care during emergency situations as are currently being witnessed in the zone. We failed to sample women from the Southeast zone to balance the representation and ensure that equal hospitals and women from the northern and southern parts of the country were represented in the sample. Thus, the results of the study are generalizable to the entire country and can be interpreted for policymaking at the national and subnational levels. However, one major limitation of this study is the retrospective study design. Because of the poor data archival and retrieval systems in many hospitals in Nigeria, it is likely that retrospective data collection will be fraught with errors. To overcome this limitation, we used a double data collection method in which two sets of data collectors obtained data from each of the hospitals. The data were further cross-checked with the existing hospital records (in the Records Departments of the Hospitals), and specific case records were reviewed when discrepancies existed. Thus, we believe that the data are substantially accurate and are useful for investigating and resolving the research question.

## Conclusions

We conclude that the maternal mortality ratios in Nigeria’s referral hospitals are worsened by high client provider ratios, with few providers attending a large number of pregnant women experiencing complications. This situation is more severe in General Hospitals (first referral hospitals) compared to Teaching Hospitals (second referral hospitals). Efforts to improve the quantum of maternal healthcare providers, especially at the first referral level, and build their capacity to deliver effective care represents a critical intervention for reducing the currently high rate of maternal mortality in Nigeria.

## Abbreviations

ANC: Antenatal care
BEmOC: Basic Emergency Obstetric Care
CEmOC: Comprehensive Emergency Obstetric Care
FMOH: Federal Ministry of Health
MMR: Maternal Mortality Ratio
MNCH: Maternal Newborn Child Health
NHREC: National Health Research Ethics Committee of Nigeria
PHC: Primary Health Care
WHARC: The Women’s Health and Action Research Centre
WHO: World Health Organization
